# T cell epigenetic remodeling and accelerated epigenetic aging are linked to long-term immune alterations in childhood cancer survivors

**DOI:** 10.1186/s13148-018-0561-5

**Published:** 2018-11-06

**Authors:** Sara Daniel, Vibe Nylander, Lars R. Ingerslev, Ling Zhong, Odile Fabre, Briana Clifford, Karen Johnston, Richard J. Cohn, Romain Barres, David Simar

**Affiliations:** 1 Mechanisms of Disease and Translational Research, School of Medical Sciences, UNSW Sydney, Wallace Wurth Building East Room 420, Sydney, NSW 2052 Australia; 2 The Novo Nordisk Foundation Center for Basic Metabolic Research, Faculty of Health and Medical Sciences, Panum, University of Copenhagen, 2200 Copenhagen N, Denmark; 3 Bioanalytical Mass Spectrometry Facility, Mark Wainwright Analytical Centre, UNSW Sydney, Sydney, Australia; 4 School of Women’s and Children’s Health, UNSW Sydney and Kids Cancer Centre, Sydney Children’s Hospital Network, Randwick, Australia

**Keywords:** Epigenetic aging, DNA methylation, inflammation, cancer survivors, T cell

## Abstract

**Background:**

Cancer treatments have substantially improved childhood cancer survival but are accompanied by long-term complications, notably chronic inflammatory diseases. We hypothesize that cancer treatments could lead to long-term epigenetic changes in immune cells, resulting in increased prevalence of inflammatory diseases in cancer survivors.

**Results:**

To test this hypothesis, we established the epigenetic and transcriptomic profiles of immune cells from 44 childhood cancer survivors (CCS, > 16 years old) on full remission (> 5 years) who had received chemotherapy alone or in combination with total body irradiation (TBI) and hematopoietic stem cell transplant (HSCT). We found that more than 10 years post-treatment, CCS treated with TBI/HSCT showed an altered DNA methylation signature in T cell, particularly at genes controlling immune and inflammatory processes and oxidative stress. DNA methylation remodeling in T cell was partially associated with chronic expression changes of nearby genes, increased frequency of type 1 cytokine-producing T cell, elevated systemic levels of these cytokines, and over-activation of related signaling pathways. Survivors exposed to TBI/HSCT were further characterized by an Epigenetic-Aging-Signature of T cell consistent with accelerated epigenetic aging. To investigate the potential contribution of irradiation to these changes, we established two cell culture models. We identified that radiation partially recapitulated the immune changes observed in survivors through a bystander effect that could be mediated by circulating factors.

**Conclusion:**

Cancer treatments, in particular TBI/HSCT, are associated with long-term immune disturbances. We propose that epigenetic remodeling of immune cells following cancer therapy augments inflammatory- and age-related diseases, including metabolic complications, in childhood cancer survivors.

**Electronic supplementary material:**

The online version of this article (10.1186/s13148-018-0561-5) contains supplementary material, which is available to authorized users.

## Background

Improvement in the cure rate of childhood cancer, and in particular acute lymphoblastic leukemia, has led to a significant increase in the number of long-term survivors [1]. However, more than 95% of childhood cancer survivors (CCS) develop chronic health conditions by the age of 45 [2]. The most frequently reported complications include cardiac, endocrine, or neurological conditions [3], chronic diseases that all share an inflammatory component. While a combination of cancer treatment, lifestyle and environmental factors could contribute to the development of these chronic conditions [4, 5], we and others have identified total body irradiation (TBI) and hematopoietic stem cells transplant (HSCT) as major risk factors for the development of metabolic, cardiovascular, and other health conditions in childhood cancer survivors [6, 7]. When compared to chemotherapy alone, exposure to radiation resulted in a higher cumulative incidence of several chronic health conditions 20 years post-diagnosis, with TBI being the mode of radiation causing the highest incidence of severe conditions [3]. We further reported in a cohort of 248 CCS that the prevalence of metabolic complications was more than doubled at a median of 12.7 years after remission [7]. More recently, we have established in a mouse model that irradiation in the absence of HSCT led to altered muscle and adipose lineage commitment and metabolic dysfunction later in life [8].

Immune disturbances contribute to cardiovascular, neurological, and metabolic conditions, notably in early development of these diseases [9, 10]. Radiation-induced immune response, including macrophage activation, neutrophil, and lymphocyte recruitment, leads to the production of pro-inflammatory mediators to support anti-tumor activity and clearance of apoptotic cells [11, 12], with a similar response having been described in response to chemotherapy [13]. Although this inflammatory process is usually confined to the acute phase of the radiation response [14], long-term immune changes could also persist following irradiation, chemotherapy, or HSCT [15, 16]. As there is still limited evidence to support the existence of long-term chronic inflammation, the mechanisms responsible for long-term chronic immune disturbances in cancer survivors and their potential consequences on survivors’ health still remain unknown.

We hypothesized that childhood cancer survivors, in particular when exposed to TBI/HSCT, present long-lasting immune alterations that could contribute to diseases later in life. Our results show that exposure to TBI/HSCT is associated with persistent chronic inflammation, a remodeling of immune cells epigenome and altered immune functions. We further establish that T cell from childhood cancer survivors presents an epigenetic signature consistent with accelerated aging. We propose that persistent alterations of the immune system in childhood cancer survivors contributes to higher risk of secondary diseases, including cardio-metabolic diseases.

## Results

To study the long-term effect of cancer treatment on immune functions, we recruited childhood cancer survivors who had received chemotherapy alone or in combination with TBI/HSCT in the course of their treatment, with clinical characteristics and cancer history presented in Table 1. The majority of our population was composed of survivors of acute lymphoblastic or myeloid leukemia, as well as Hodgkin’s and non-Hodgkin’s lymphoma, and all survivors received chemotherapy. Our two groups were age-matched, with no difference in age at inclusion, at diagnosis, or in number of years on remission (Table 1). Survivors in the TBI/HSCT group tended to be shorter and lighter (*p* = 0.05 and *p* = 0.07 respectively).

**Table 1:** Participants’ characteristics

	Non-IRR	TBI/HSCT	*p* value (TBI/HSCT vs non-IRR)
Number of participants	30	14	
Sex (male/female)	15/15	9/5	0.25
Age (year, range)	22.1 (16–34)	25.1 (16–39)	0.48
Age at diagnosis (year, range)	7.2 (0.2–17.7)	7.7 (1.2–16.6)	0.78
Height (cm)	170 ± 9	163 ± 12	0.05
Weight (kg)	73.9 ± 18.8	62.0 ± 19.3	0.07
Body mass index (kg/m^2^)	25.5 ± 5.7	23.2 ± 6.8	0.26
Waist-to-height ratio	50.8 ± 8.5	51.4 ± 11.1	0.84
Diagnosis (number of survivors, %)			
Acute lymphoblastic leukemia	12 (40%)	5 (36%)	
Relapse	0 (0%)	1 (7%)	
Acute myeloid leukemia	1 (3%)	6 (43%)	
Relapse	0 (0%)	1 (7%)	
Neuroblastoma	1 (3%)	2 (14%)	
Malignant histiocytosis	0 (0%)	1 (7%)	
Hodgkins lymphoma	2 (13%)	0 (0%)	
Non-Hogkins lymphoma	3 (10%)	0 (0%)	
Other type of cancer	11 (21%)	0 (0%)	
Treatments (number of survivors, %)			
Chemotherapy	30 (100%)	14 (100%)	
TBI	0 (0%)	14 (100%)	
Hematopoietic stem cell transplant	3 (10%)	14 (100%)	
Triglycerides (mmol/l)	1.03 ± 0.50	2.11 ± 2.08	0.01
Total cholesterol (mmol/l)	4.82 ± 0.79	5.14 ± 1.12	0.28
LDL-cholesterol (mmol/l)	2.86 ± 0.53	2.97 ± 1.13	0.67
HDL-cholesterol (mmol/l)	1.59 ± 0.71	1.17 ± 0.41	0.05
Glucose (mmol/l)	4.74 ± 0.47	6.04 ± 4.19	0.10
Insulin (mIU/l)	7.17 ± 5.76	11.3 ± 8.60	0.07
HOMA-IR	0.96 ± 0.73	1.84 ± 1.98	0.04

*LDL* low-density lipoprotein, *HOMA-IR* homeostatic model assessment insulin resistance index, *Non-IRR* non-irradiated, *TBI* total body irradiation, *HSCT* hematopoietic stem cell transplant

### TBI/HSCT is associated with long-term epigenetic remodeling in T cell

**Fig. 1:**
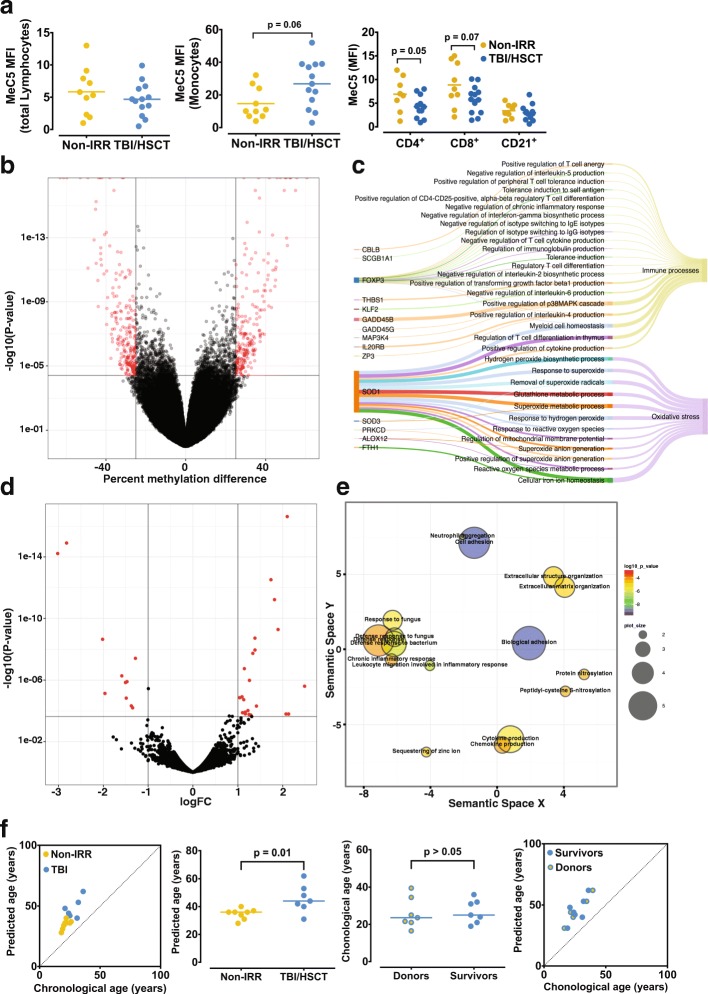
Epigenetic and transcriptomic analysis of T cell in childhood cancer survivors (CCS). **a**. No difference in global DNA methylation was observed in total lymphocytes or B cells (CD3^−^CD21^+^) between CCS who received total body irradiation and hematopoietic stem cell transplant (TBI/HSCT) compared to those who received no irradiation (non-IRR, *p* > 0.05). CD4^+^ cells from CCS treated with TBI/HSCT showed global hypomethylation (*p* = 0.05), with a similar trend in CD8^+^ cells (*p* = 0.07), whereas monocytes tended to show hypermethylation (*p* = 0.06). **b**. Volcano plot representing the distribution of differentially methylated genes in the two groups of survivors, with more than 400 genes differentially methylated (represented in red, with a difference in methylation of more than 25%). **c**. Sankey diagram showing representative Gene Ontology (GO) terms clusters from differentially methylated genes, including all main GO terms related to immunological processes or oxidative stress. The width of the arrow is proportional to the contribution of the gene to the GO term and to the contribution of the GO term to immunological processes or oxidative stress **d**. Volcano plot reflecting the distribution of differentially expressed genes (in red) between the two groups of CCS. **e**. Representative GO terms clusters from differentially expressed genes showed a particular enrichment in GO terms related to immune processes and oxidative stress. **f**. Predicted biological age based on methylation levels on three specific CpG sites on *ASPA*, *ITGA2B*, and *PDE4C* was higher in CCS treated with TBI/HSCT. Both groups showed increased distance from the regression line, suggesting increased biological predicted age compared to chronological age. Chronological age was not different between the donors and the children who received hematopoietic stem cells transplant at the time of the experiment (*p* > 0.05)

Reports have suggested that inflammation could persist years after cancer treatments [17], potentially through a long-term “memory” of the treatment, implying epigenetic mechanisms. Thus, we assessed the DNA methylation profile of immune cells. CD4^+^ (*p* = 0.05) and CD8^+^ T cell (*p* = 0.07) tended to show lower global DNA methylation levels in CCS treated with TBI/HSCT, while monocytes tended to show higher methylation levels (*p* = 0.06), with no difference in other populations (Fig. 1a). We then tested if these changes in methylation were gene-specific and performed reduced representation bisulfite sequencing (RRBS) in CD4^+^ T cell. We found differential methylation at cytosines which corresponded to 419 different neighboring genes in the TBI/HSCT group (Fig. 1b, Additional file 1: Table S1). Out of the 419 differentially methylated regions (DMRs), 9 were identified in enhancers and 47 in promoters (Additional file 1: Table S1). Differential methylation was also found at microRNA (miRNA) loci (Additional file 1: Table S1), including miR-942 and miR-378c, which have been implicated in acute response to irradiation, or miR-124 which is linked to T cell polarization and inflammation [18, 19]. Using GO analysis on our 419 coding genes containing differentially methylated cytosines, we identified enrichments for GO terms linked to oxidative stress (15/77 GO terms found in our data, Fig. 1c, Additional file 1: Table S2) and genes related to immune processes (21/77 terms) including *positive* and *negative regulation of cytokines production* (Fig. 1c, Additional file 1: Table S2). Transcriptomic analysis of CD4^+^ T cell by RNA-seq returned a small subset of genes differentially expressed (29, Fig. 1d, Additional file 1: Table S3a). Although we did not detect differential methylation at the proximity of the differentially expressed genes, except for *adhesion G protein-coupled receptor G1* (also known as GPR56), we found enrichment for GO terms related to inflammation, similar to the DMR-associated genes (Fig. 1e, Additional file 1: Table S3B). Collectively, these results support that TBI/HSCT stably alters the epigenome of T cell.

### Cancer survivors treated with TBI/HSCT show long-term altered immune function

**Fig. 2:**
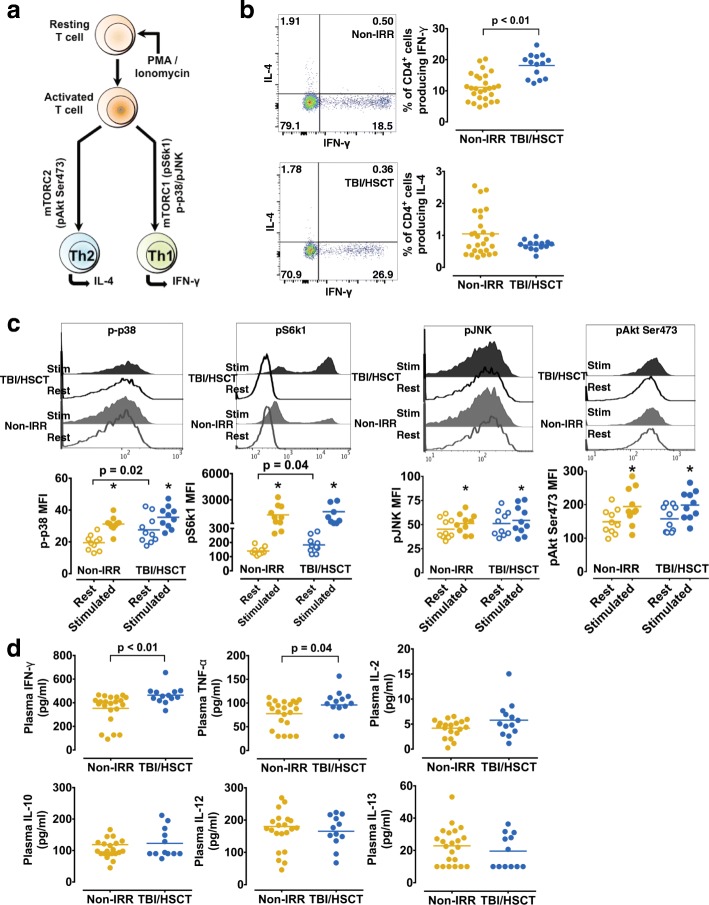
CD4^+^ T cell is altered in childhood cancer survivors (CCS) treated with TBI (total body irradiation)/HSCT (hematopoietic stem cell transplant). **a**. Schematic representation of intracellular signaling involved in polarized activation in T cells. **b**. Representative FACS dot plot of CD4^+^ cells producing interferon (IFN)-γ (X-axis) or interleukin (IL)-4 (Y-axis) in CCS treated with TBI/HSCT (bottom) or no irradiation (non-IRR, top). The frequency of CD4^+^ cells producing IFN-γ was higher in CCS treated with TBI/HSCT compared to non-IRR CCS (*p* < 0.01), with no difference in CD4^+^ cells producing IL-4 (*p* = 0.3). **c**. Representative histograms for phosphorylated p38, phosphorylated ribosomal protein S6 kinase 1 (pS6 k1), phosphorylated c-Jun N-terminal kinase (pJNK), and phosphorylated Akt on Ser473 under resting condition (Rest) and upon mitogen stimulation (Stim). Upon mitogen stimulation, p-p38, pS6k1, pJNK, and pAkt Ser473 were all significantly increased (*p < 0.05, compared to resting condition) in CD4^+^ cells in both groups (*n* = 10 in each group). Higher resting phosphorylation levels of p38 and S6 k1 were observed in CD4^+^ cells from CCS treated with TBI (*p* = 0.02 and *p* = 0.04 respectively). **d**. Plasma levels of Th1 cytokines including interferon (IFN)-γ and tumor necrosis factor (TNF)-α were significantly elevated in CCS treated with TBI/HSCT (*p* < 0.01 and *p* = 0.04 respectively) whereas interleukin (IL)-10 and IL-13, or IL-2 and IL-12, did not differ between the two groups (*p* > 0.05)

To determine the consequences of the remodeling of the T cell epigenome, we analyzed T cell function (Fig. 2a). In mitogen-stimulated CD4^+^ and CD8^+^ T cell from the TBI/HSCT group, we found a greater frequency of interferon (IFN)-γ but not interleukin (IL)-4-producing cells (Fig. 2b, Additional file 1: Figure S1a). Intracellular signaling pathways, including mTOR (mammalian target of rapamycin) and MAPK (mitogen-activated protein kinases), are critical regulators of T cell polarization and cytokines production (Fig. 2a). Quantification of p38, ribosomal protein S6 kinase1 (S6 k1), c-Jun N-terminal kinase (JNK) and Akt phosphorylation in CD4^+^ and CD8^+^ T cell showed a marked increase for all four proteins in both groups upon mitogen stimulation (Fig. 2c, Additional file 1: Figure S1b). However, the TBI/HSCT group showed over-activation of p38 and S6 k1 under resting conditions in CD4^+^ (Fig. 2c) and CD8^+^ cells (Additional file 1: Figure S1b), potentially linked to the increased frequency of type 1 cytokine-producing T cell. These functional changes in T cell from the TBI/HSCT group were associated with elevated systemic levels of IFN-γ and tumor necrosis factor (TNF)-α (*p* < 0.05, Fig. 2d), suggesting a link between radiation, HSCT, and long-term inflammation.

**Fig. 3:**
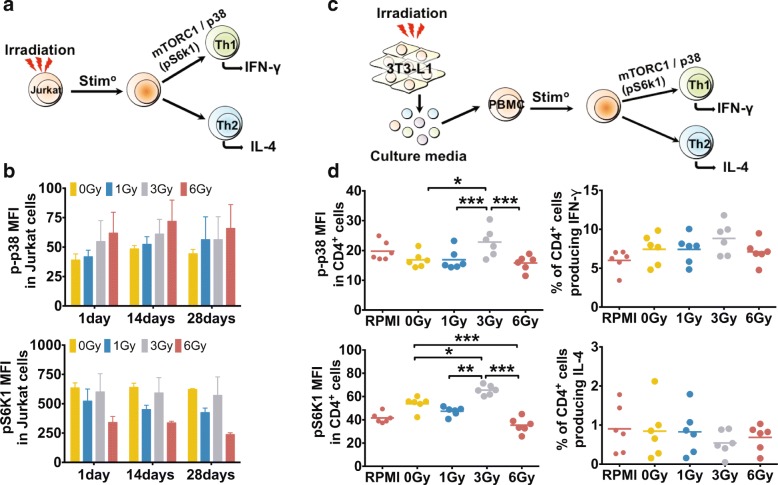
Intracellular signaling pathways involved in T cell polarization are activated by irradiation through a bystander mechanism. **a**. Schematic representation of the in vitro model to investigate the direct effect of irradiation on a T cell line (Jurkat cells). **b**. No effect of irradiation (0 to 6Gy) was observed on the activation of p38 or ribosomal protein S6 kinase 1 (S6 k1) in Jurkat cells (*p* > 0.05). **c**. Schematic representation of the co-culture model used to investigate the indirect effect of irradiation on the activation of intracellular signaling pathways involved in T cell polarization. **d**. In CD4^+^ cells, pre-incubation with culture media obtained from irradiated adipocytes with a dose of 3Gy resulted in higher phosphorylation levels of both p38 and S6 k1 (*p < 0.05, **p < 0.01, ***p < 0.001). No effect of the pre-incubation in culture media from irradiated adipocytes was observed on the percentage of CD4^+^ producing interferon (IFN)-γ or interleukin (IL)-4 although a trend towards an overall effect was observed on the frequency of IFN-γ producing CD4^+^ cells (*p* = 0.12)

**Fig. 4:**
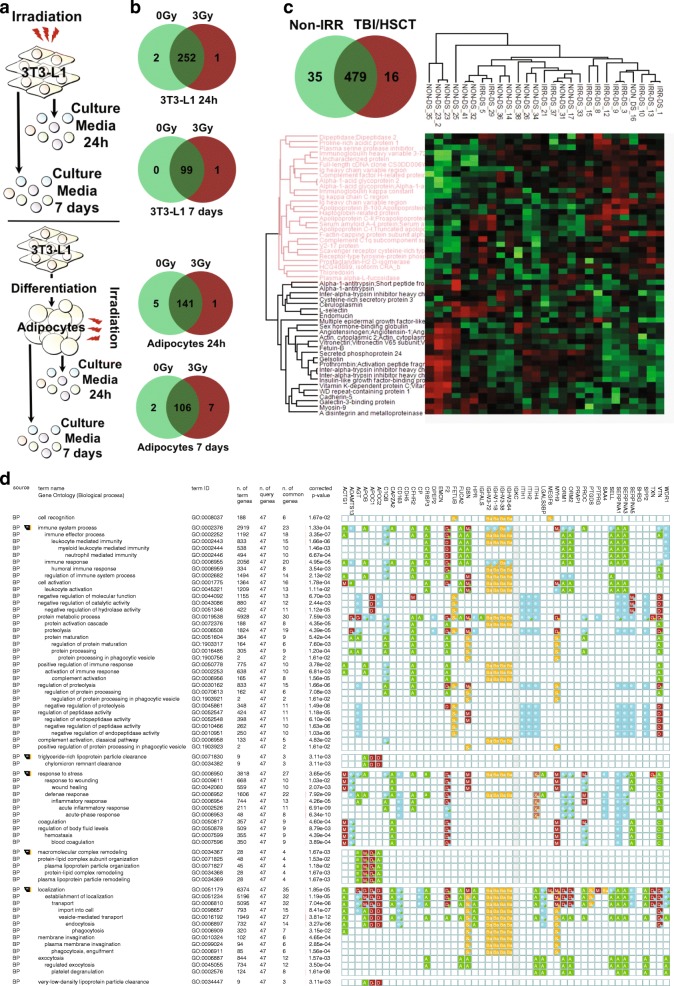
Circulating factors involved on the indirect effect of irradiation on T cell function **a**. Schematic representation of the in vitro model to identify circulating factors responsible for the indirect effect of irradiation on T cell function. **b**. Venn diagram representing uniquely identified proteins in culture media from non-irradiated cells (0Gy, light green), from irradiated cells (3Gy, red), or in both conditions (dark green), in four different conditions (undifferentiated and differentiated fibroblasts at 24 h and seven days post-irradiation). **c**. Venn diagram representing uniquely identified proteins in plasma from non-irradiated (non-IRR, light green) or TBI/HSCT-treated survivors (TBI/HSCT, red), or found in both groups (dark green). Unsupervised hierarchical cluster analysis of protein abundance identifying specific patterns of proteins abundance profile in the two groups of survivors. **d**. Gene ontology analysis performed on proteins showing a different pattern of abundance between the two groups of survivors. S: Sequence or structural similarity [ISS]; Ba: biological aspect of ancestor [IBA]; A: traceable author [TAS]; e: electronic annotation [IEA]; D: direct assay [IDA]; M: mutant phenotype [IMP]; G: genetic interaction [IGI]; a: non-traceable author [NAS]; X: expression pattern [IEP]

Acute exposure to radiation triggers a transient stress response resulting in pro-inflammatory cytokines production [14]. Thus, we tested the contribution of direct irradiation to T cell polarization and MAPK/mTOR activation using an in vitro model (Fig. 3a). Direct irradiation did not affect p-p38 or pS6 k1 (*p* > 0.05, Fig. 3b), or pJNK, pAkt-Ser473, and the frequency of IL-4/IFN-γ-producing cells in Jurkat cells (Additional file 1: Figure S2a-b). We then investigated the indirect effect of irradiation by exposing mitogen-stimulated PBMCs collected from healthy donors to the conditioned media obtained from irradiated fibroblasts (Fig. 3c). We found that conditioned media from fibroblasts irradiated with a dose of 3Gy increased phosphorylation of p38 and S6 K1 in CD4^+^ cells (Fig. 3d) but had no effect on pJNK or pAkt-Ser473 (Additional file 1: Figure S2c). Exposure to conditioned media from fibroblasts irradiated with a dose of 3Gy also had no effect on the frequency of CD4^+^ cells or CD8^+^ cells producing IFN-γ (*p* = 0.14, Fig. 3d) or IL-4 (*p* > 0.05, Fig. 3d, Additional file 1: Figure S3a-b for CD8^+^ cells). These results suggest that irradiation may exert its effect on T cell through secreted factors. To identify proteins secreted by irradiated cells, we analyzed cell culture supernatants from irradiated (1) fibroblasts or (2) fully differentiated adipocytes at 24 h and 7 days post-exposure using mass spectrometry (Fig. 4a). Most detected proteins were secreted in both irradiated or non-irradiated conditions (Fig. 4b), with some proteins uniquely detected in the irradiated or non-irradiated conditions, although this did not reach statistical significance (Additional file 1: Table S4). We next analyzed plasma samples from CCS and detected more than 50 proteins with a distinct expression pattern (Fig. 4c). GO analysis identified a specific enrichment for pathways related to *cell stress*, *immune processes* and *inflammation* (Fig. 4d). Of interest, the alpha-1-antitrypsin protein (encoded by the *SERPINA1* gene) was decreased in the TBI/HSCT group. Given the role of alpha-1 antitrypsin in the inhibition of pro-inflammatory cytokines secretion [20], decreased levels of this protein could participate in the immune changes in CCS treated with TBI/HSCT. Our cluster analysis suggested that the expression pattern of these proteins was group-specific. Collectively, our data suggest that secreted factors participate in a long-term alteration of the immune system in survivors exposed to irradiation and hematopoietic stem cell transplant.

### Childhood cancer survivors are characterized by accelerated epigenetic aging

Given that T cell epigenetic remodeling specifically affected genes involved in inflammation and oxidative stress, two processes associated with aging, we investigated the contribution of epigenetic factors in cancer treatment-induced accelerated aging. Using the Epigenetic-Aging-Signature method [21], we found that both groups of survivors had increased predicted biological age (Fig. 1f), with CCS treated with TBI/HSCT showing a higher predicted biological age (*p* = 0.01, Fig. 1f). Moreover, the chronological age was correlated to the predicted age in non-irradiated CCS (*n* = 8, *r* = 0.85, *p* < 0.01, Fig. 1f) but not in the TBI/HSCT group (*n* = 7, *p* > 0.05, Fig. 1f). Since all survivors treated with TBI also received HSCT, our results could indicate that the predicted biological age reflect the age of the donor as previously suggested [21]. However, we did not find any difference between the donors’ and the recipients’ chronological age (Fig. 1f). In addition, the inclusion of the donors’ age in the model did not change the correlation between chronological and predicted age (Fig. 1f), suggesting that TBI/HSCT could be responsible for the accelerated epigenetic aging in T cell. When we compared the methylation levels on three specific CpG sites used to establish the Epigenetic-Aging-Signature, we found a hypermethylation only on *PDE4C* in the TBI/HSCT group (*p* > 0.05 for *ASPA* and *ITGA*, *p* < 0.01 for *PDE4C*, Additional file 1: Figure S1c). Consistent with the Epigenetic-Aging-Signature, transcriptomic analysis of T cells showed that more than 10% of differentially expressed genes belonged to the “innate and adaptive immunity, cytokine, and chemokine” cluster of genes, which constitutes genes previously reported to contribute to the aging signature of peripheral blood (Additional file 1: Table S3A) [22]. The present results support a role for cancer treatment in the accelerated aging of T cell in cancer survivors.

Multiple reports have suggested an increased prevalence of inflammatory diseases in cancer survivors. We thus investigated the presence of cardio-metabolic risk factors in our cohort. Consistent with previous studies [3, 7], CCS treated with TBI/HSCT had impaired metabolic profile, including increased triglycerides and reduced high-density lipoprotein cholesterol (HDL-C) levels (*p* < 0.05, Table 1). Importantly, these changes were not due to a difference in age, age at diagnosis, body mass index (BMI), or waist-to-hip ratio (*p* > 0.05, Table 1). The level of insulin resistance was also higher in the TBI/HSCT group (*p* = 0.04, Table 1), while fasting blood glucose and insulin levels also tended to be higher in that group (*p* = 0.1 and *p* = 0.07 respectively; Table 1). These results confirm that CCS, in particular those who received TBI/HSCT, are at increased cardio-metabolic risks with a potential link with chronic inflammation.

## Discussion

Our results suggest that cancer treatment stably remodels the T cell epigenome at specific genes controlling inflammation and oxidative stress in CCS. Using various markers of immune cell function and signaling pathways regulating pro-inflammatory cytokines production, we establish a link between T cell epigenetic remodeling and low-grade inflammation in survivors, in particular those who received TBI/HSCT. We identified that TBI/HSCT is associated with accelerated aging and propose a role for epigenetic changes in the elevated risk of age- and inflammation-related diseases long after treatment in CCS [23].

We found that CCS treated with TBI/HSCT are characterized by systemic inflammation. The secretion of cytokines by immune cells is regulated by epigenetic mechanisms [24]. Our global DNA methylation analysis of T cell from survivors supports a dramatic remodeling of the epigenome after TBI/HSCT. Radiation-induced long-term epigenetic changes have been reported in cultured human keratinocytes and rodents [25, 26] and could mediate the imprinting of a long-term “memory” of the radiation insult, potentially affecting immune cell function. HSCT has also been associated with an inflammatory response, although limited information regarding its long-term effect has been reported [16]. Nonetheless, it can be hypothesized that even sub-clinical levels of graft-versus-host disease could have contributed to the immune changes reported here. We have previously shown that the epigenome of lymphocytes is linked to environmental influences, notably nutritional [27, 28]. Epigenetic mechanisms including changes in DNA methylation, histone modifications, or even miRNAs are well-described as modulators of immune cell differentiation and function [29, 30]. For example, differentiation and polarized activation of T helper or cytotoxic cells are controlled by global DNA methylation at particular gene loci [31, 32]. Although we did not observe specific changes at previously reported gene loci, we identified several differentially methylated genes involved in critical pathways controlling T cell differentiation or cytokine production. It cannot be ruled out that by investigating epigenetic changes in global CD4^+^ T cell, we might have missed DNA methylation changes specific to effector, memory, or regulatory T cell [31, 32]. In addition, these T cell subtypes present different epigenetic signature [31, 32]. Thus, our results could also suggest changes in the distribution of T cell subsets.

We found a greater frequency of type 1 cytokine-producing T cell in irradiated survivors, with these cells showing higher activation of p38 MAP kinase pathway and mTOR Complex 1 (mTORC1) signaling (S6 k1), potentially through a bystander effect. Direct and indirect exposure to ionizing radiation activate MAPKs (p38/JNK) and the PI3K (phosphoinositide 3-kinase)/Akt/mTOR pathways [12, 33], and both p38 and mTORC1 are critical for Th1 polarization and type 1 cytokines production [34, 35]. The over-activation of both p38 and mTORC1 is consistent with the greater frequency of Th1 cells and the higher levels of pro-inflammatory cytokines in survivors who received TBI/HSCT. The response to radiation involves the mobilization of immune cells and pro-inflammatory cytokine production, although these immune changes usually resolve within hours to days upon exposure [36]. Similarly, HSCT can be associated with an inflammatory response, though the long-term consequences remain unclear [16]. Delayed or chronic immune activation after radiation has been reported in atomic bomb survivors more than 50 years after exposure [37]. Impaired immune function has also been reported up to 10 years following radiation in long-term cancer survivors [15], although in both cases, the molecular mechanisms were not identified. Our results suggest that such immune changes could result from an indirect effect of irradiation, potentially due to circulating factors. This bystander effect has been previously suggested as playing a key role in the adaptations following irradiation [12]. Although we could not identify specific factors driving the epigenetic remodeling and altered immune function, it is likely that such factors might contribute to these phenomena [12]. We can speculate that the observed changes in the T cell methylome and T cell function could be involved in the perpetuation of the cancer treatment-induced pro-inflammatory programming. Limitations associated with our in vitro model to investigate the direct effect of irradiation, in particular the absence of the regulator of the Akt/mTOR pathway Phosphatase and TENsin homolog (PTEN) in Jurkat cells, forces us to caution when interpreting our results using this cell system. Further work is needed using other cell models to fully clarify the influence of irradiation on the epigenome.

Inflammation and oxidative stress are associated with several processes involved in aging [38]. Recently, changes in both the epigenome and the transcriptome of circulating leukocytes have been linked to the biological (rather than chronological) age of an individual [22, 39]. A marked remodeling of the epigenome encompassing global DNA hypomethylation and gene-specific hypermethylation at particular loci has been reported during aging [38], consistent with the hypomethylation we observed in T helper cells at the global DNA level. At the gene level, particular loci or even CpG sites have been identified as predictive markers of biological age [39] and allowed us to identify an increased predicted biological age in cancer survivors. Several components of the radiation response can contribute to accelerated aging or inflammatory diseases, including low-grade systemic inflammation, activation of MAPK/mTOR signaling, epigenetic remodeling, or oxidative stress [12, 33, 38, 40, 41]. In the present study, we identified the presence of such disturbances in cancer survivors more than 10 years post-treatment, which potentially explains the accelerated aging reported in this population [23]. We and others have previously shown that both reactive oxygen species and pro-inflammatory cytokines can affect DNA methylation [42, 43]. We can speculate that the initial inflammatory response and oxidative burst in response to treatment might have contributed to the epigenetic remodeling of immune cells, although the timing and nature of the events that might have led to this “long-term memory” are still to be unraveled. These changes in the T cell methylome might be involved in the perpetuation of the pro-inflammatory programming contributing to the early onset of cardio-metabolic complications in cancer survivors.

## Conclusions

Improvements in childhood cancer survival are associated with higher prevalence of treatment-associated diseases later in life, notably of age-related conditions including cardiometabolic diseases. We identified low-grade inflammation and altered immune cell function in survivors treated with TBI/HSCT. This was associated with a dramatic remodeling of the T cell epigenetic signature and features of accelerated epigenetic aging. These immune changes could account for the increased risk of inflammatory and age-associated diseases later in life. A greater understanding of the mechanisms by which cancer treatments affect the epigenome of immune cells may help to decrease long-term complications in cancer survivors.

## Methods

### Participants

We recruited 44 childhood cancer survivors, diagnosed between 0 and 18 years, in full remission for more than 5 years and 16 years old and older at the time of enrolment. Survivors who had experienced graft-versus-host disease were not included in this study. This project was approved by the Human Research Ethics Committee (UNSW Sydney, HREC-10/017) and performed in accordance with Helsinki Declaration procedures. The nature of the project was explained and participants provided informed consent. Participants had received chemotherapy only (non-IRR) or a combination of chemotherapy with TBI and HSCT (TBI/HSCT). Anthropometric data and medical history were recorded and BMI calculated.

### Biological analyses

Fasting blood samples were collected and stored at − 80 °C. Serum insulin (Immulite 2000, Diagnostic Products Corporation, CA, USA), glucose levels, HDL-C, low-density lipoprotein cholesterol (LDL-C), and triglycerides were measured (LX20 analyzer, Beckman Coulter, CA, USA), and the insulin resistance index was calculated (Homeostasis Model Assessment, HOMA-IR) [44]. Systemic cytokine levels including IL-2, IL-4, IL-5, IL-10, IL-12, IL-13, IFN-γ, TNF-α, and granulocyte-monocyte colony stimulating factor were measured (Bio-Plex Pro Human Cytokine Th1/Th2 Assay, Bio-Rad Laboratories Inc., Australia, Intra-assay CV: 7–12% and Inter-assay CV: 6–10%). Results were reported only for cytokines that were detectable in more than 30% of the participants.

### Flow cytometry

Peripheral blood mononuclear cells (PBMCs) were isolated from whole blood and cryopreserved [28]. PBMCs were thawed, left to recover overnight, and mitogen-stimulated for 6 h (2 μg/ml phorbol 12-myristate 13-acetate/ionomycin, Sigma-Aldrich, Australia), with the addition of monensin (BioLegend, CA, USA) after 2 h. PBMCs were then fixed (2% paraformaldehyde), stained for dead cells (ethidium monoazide bromide, Sigma-Aldrich), and permeabilized in ice-cold methanol. Cells were then stained using CD4-V500, CD-PE-Cy7, IL-4-PE, IFN-γ-Pacific blue (BD Biosciences, Australia), and phosphorylated pAkt-Ser473-AF647 (BD Biosciences), pS6k1-AF488, pJNK-AF488, and phosphorylated p38-AF647 (Cell Signaling Technology, MA, USA). PBMCs were analyzed on FACS CantoII (BD Biosciences), with data post-compensated and analyzed using FlowJo (Tree Star Inc., OR, USA). Results were expressed as percentage of IL-4^+^/IFN-γ^+^ cells or normalized median fluorescence intensity for pAkt, pS6k1, pJNK, and p-p38 in CD4^+^ and CD8^+^ cells.

### In vitro and co-culture models

Jurkat cells (Clone E6–1, ATCC, VA, USA) were irradiated with doses of 0, 1, 3, or 6Gy (Xrad 320, Precision X-ray Inc., CT, USA) and left to recover for 24 h. At days 1, 14, and 28, post-irradiation cells were mitogen-stimulated and analyzed by flow cytometry as described above. Adipocytes (3 T3-L1, CL-173, ATCC) were irradiated with doses of 0, 1, 3, and 6Gy and left to recover for 24 h prior to the collection of culture media. PBMCs from six young healthy donors were mitogen-stimulated in conditioned media from irradiated adipocytes and analyzed by flow cytometry as described above.

### Protein sample preparation and mass spectrometry

Plasma samples (14ul) from childhood cancer survivors were depleted from the 14 most abundant proteins (Sigma Seppro IgY14 Spin columns). Depleted plasma sample and conditioned media from cultured fibroblasts and fibroblasts differentiated into adipocytes (only 0/3Gy and at 24 h/7 days post-irradiation) were processed as previously described with slight modifications (see “Additional file 1”) [45]. A survey scan *m*/*z* 350–1750 was acquired in the Orbitrap and lock mass enabled with data-dependent tandem MS analysis performed using a top-speed approach (cycle time of 2 s). MS^2^ spectra were fragmented by HCD activation mode and the ion trap was selected as the mass analyzer. Peak lists were generated using Mascot Daemon and submitted to Mascot (version 2.5.1, Matrix Science). Label-free quantitation was carried out using MaxQuant (version 1.5.6.5) and Perseus (version 1.5.6.0) and pathway analysis using G-Profiler.

### Epigenetic analyses

#### Global DNA methylation

Global DNA methylation was measured in PBMCs, using surface markers (CD3-PE, CD4, or CD21-APC, CD8, or CD14-PerCP, BD Biosciences) and anti-5-methylcytidine antibody (AbD Serotec, Bio-Rad) conjugated to AF488 (Zenon AF488 Mouse IgG1 labeling kit, Life Technologies, Australia) as previously described [28].

#### Gene-specific methylation

PBMCs were sorted on Influx Cell Sorter (BD Biosciences) using specific surface markers (CD4 and CD19/CD20-BV421, CD8-BV510, CD3-FITC, CD56-PE-CF594, CD45-PerCP-Cy5.5, and CD14-APC, BD Biosciences). Cells were then lysed before DNA/RNA extraction (AllPrep DNA/RNA/miRNA Universal kit on QIAcube, Qiagen, Australia). CD4^+^ T cell DNA was processed using reduced representation bisulfite sequencing with minor changes [46]. Genomic DNA was fragmented by overnight incubation with Msp1 restriction enzyme and adenylated with dATP and Klenow DNA polymerase (3′ to 5′ exo minus) before ligation to TruSeq adapters (Illumina, CA, USA). Twelve ligated samples were pooled and processed for bisulfite conversion using EZ DNA methylation kit (Zymo Research, CA, USA). Libraries were enriched by PCR, purified with AMPure beads, and sequenced on a HiSeq (50 bp single-end sequencing, Illumina, Danish National High-Throughput DNA Sequencing Centre, Denmark). Preprocessed reads were aligned to hg38 with Bismark [47], and differential methylation was analyzed with MethylKit [48]. All regions were annotated with the Bioconductor package ChIPseeker [49]. CpGs with more than 25% methylation change and *q* value < 0.01 were included for further analysis. Gene Ontology (GO) analysis was performed using a hypergeometric test on individual CpGs and scatter plots generated with REVIGO [50]. T cell-specific enhancers were downloaded from SlideBase [51, 52] and lifted to hg38 using the UCSC liftOver tool [53], before counting overlaps with DMRs.

#### Epigenetic-Aging-Signature

DNA methylation was measured on sorted CD8^+^ T cell at three specific CpG sites (*ASPA*, *ITGA2B*, and *PDE4C*) by bisulfite pyrosequencing. Beta values were then used in a multivariate model to estimate the predicted biological age as previously described [39].

#### Whole transcriptome sequencing

RNA from CD4^+^ T cell was sequenced following standard protocol (Truseq Stranded total RNA protocol, Illumina). After depletion of ribosomal RNA and fragmentation, cDNA synthesis was performed on the first strand, followed by second strand synthesis. cDNA was cleaned using AMPure beads, adenylated and then ligated to adapters. Following an additional beads cleanup, DNA fragments were enriched by PCR and subjected to a final beads cleanup. Libraries were sequenced on a HiSeq (100 bp single-end sequencing, Illumina, Danish National High-Throughput DNA Sequencing Centre). Reads were aligned to hg38 using Rsubread aligner, and the number of reads aligning with Gencode (version 23) was counted with featureCounts [54]. Genes with less than 1 RPKM in half the samples were excluded. Differential expression was calculated with edgeR [55] using glmFit/glmLRT functions and tagwise dispersion, with correction for multiple testing using the Benjamini–Hochberg procedure. GO analysis was performed using GOrilla [56], and scatter plots were generated with REVIGO [50].

#### Statistical analysis

Data are expressed as mean +/− standard deviation and analyzed using SPSS Statistics (IBM, NY, USA). Normality of the distribution was tested using the Skewness and Kurtosis tests and anthropometric data, biological markers, cytokines levels, T cell polarization, and global DNA methylation or predicted age were compared using Student *t* test or Mann-Whitney *U* test. A chi-square test was used to compare the frequencies between the two groups. The effect of irradiation/mitogen stimulation on intracellular signaling was tested using two-way ANOVA for repeated measures or a combination of Friedman’s ANOVA and Mann-Whitney *U* test. Correlation levels were tested using the Pearson or Spearman tests.

## Additional file

Additional file 1: **Figure S1.** CD8+ cells polarized activation in childhood cancer survivors (CCS). **Figure S2.** Intracellular signaling pathways involved in T cell polarization in response to direct and indirect irradiation. **Figure S3.** Intracellular signaling pathways and polarized activation in CD8+ cells exposed to conditioned media from irradiated adipocytes. **Table S1.** Differentially Methylated Genes. **Table S2.** Gene Ontology from differentially methylated genes. **Table S3.** a. Genes differentially expressed. b. Gene Ontology terms from differentially expressed genes. **Table S4.** Proteins identified in the supernatant of irradiated fibroblasts. (ZIP 4590 kb)
