# Supplementary material for: T cell epigenetic remodeling and accelerated epigenetic aging are linked to long-term immune alterations in childhood cancer survivors
Source: Clin Epigenetics. 2018 Nov 6;10:138. doi: 10.1186/s13148-018-0561-5 (PMC6219017; doi:10.1186/s13148-018-0561-5)
Supplement: Supplementary file 1 — Figure S1. CD8+ cells polarized activation in childhood cancer survivors (CCS). Figure S2. Intracellular signaling pathways involved in T cell polarization in response to direct and indirect irradiation. Figure S3. Intracellular signaling pathways and polarized activation in CD8+ cells exposed to conditioned media from irradiated adipocytes. Table S1. Differentially Methylated Genes. Table S2. Gene Ontology from differentially methylated genes. Table S3. a. Genes differentially expressed. b. Gene Ontology terms from differentially expressed genes. Table S4. Proteins identified in the supernatant of irradiated fibroblasts. (ZIP 4590 kb) [file 13148_2018_561_MOESM1_ESM.zip › Additional_Files_ClinicalEpigenetics_SDaniel_revised.docx]

**Supplementary Methods**

**Protein sample preparation and mass spectrometry**

Plasma samples (14ul) from childhood cancer survivors were depleted from the 14 most abundant proteins using Sigma Seppro IgY14 Spin columns and following manufacturer’s instructions. Depleted plasmas sample and conditioned media from cultured fibroblasts and fibroblasts differentiated into adipocytes (only 0 and 3Gy and at 24h and 7 days post-irradiation) were were reduced with 5mM dithiothreitol, alkalyated with iodoacetimide followed by the tryptic digestion overnight at 37C.

Digest peptides were separated by nanoLC using an Ultimate nanoRSLC UPLC and autosampler system (Dionex, Amsterdam, Netherlands). Samples (2.5 µl) were concentrated and desalted onto a micro C18 precolumn (300 µm x 5 mm, Dionex) with H2O:CH3CN (98:2, 0.1 % TFA) at 15 µl/min. After a 4 min wash the pre-column was switched (Valco 10 port UPLC valve, Valco, Houston, TX) into line with a fritless nano column (75µ x ~15cm) containing C18AQ media (1.9µ, 120 Å Dr Maisch, Ammerbuch-Entringen Germany). Peptides were eluted using a linear gradient of H2O:CH3CN (98:2, 0.1 % formic acid) to H2O:CH3CN (64:36, 0.1 % formic acid) at 200 nl/min over 30 min. High voltage 2000 V was applied to low volume Titanium union (Valco) and the tip positioned ~ 0.5 cm from the heated capillary (T=275°C) of a Orbitrap Fusion Lumos (Thermo Electron, Bremen, Germany) mass spectrometer. Positive ions were generated by electrospray and the Fusion Lumos operated in data dependent acquisition mode (DDA).

A survey scan m/z 350-1750 was acquired in the orbitrap (resolution = 120,000 at m/z 200, with an accumulation target value of 400,000 ions) and lockmass enabled (m/z 445.12003). Data-dependent tandem MS analysis was performed using a top-speed approach (cycle time of 2s). MS2 spectra were fragmented by HCD (NCE=30) activation mode and the ion-trap was selected as the mass analyzer. The intensity threshold for fragmentation was set to 25,000. A dynamic exclusion of 20 s was applied with a mass tolerance of 10ppm .

Peak lists were generated using Mascot Daemon, and submitted to the database search program Mascot (version 2.5.1, Matrix Science). Search parameters were: Precursor tolerance 4 ppm and product ion tolerances ± 0.5 Da; Met (O) carboxyamidomethyl-Cys specified as variable modification, enzyme specificity was trypsin, 1 missed cleavage was possible and the Uniprot database was searched. Label-free quantitation was carried-out using the MaxQuant (version 1.5.6.5) and Perseus (version 1.5.6.0). Pathway analysis was performed on the open-source program G-Profiler.

**Additional Figures Legend**

**Additional Figure 1.** **CD8^+^ cells polarized activation in childhood cancer survivors (CCS)**. **a.** The frequency of CD8^+^ cells producing interferon (IFN)-γ was higher in CCS treated with total body irradiation/haematopoietic stem cell transplant (TBI/HSCT) compared to non-IRR CCS (p<0·01). No difference was observed in the percentage of CD8^+^ cells producing interleukin (IL)-4 between the two groups (p>0·05). **b**. Upon mitogen stimulation, phosphorylated p38, phosphorylated ribosomal protein S6 kinase 1 (pS6k1), phosphorylated c Jun N terminal kinase (pJNK) and pAkt Ser473 were all significantly increased (*p<0·05, compared to resting condition) in CD8^+^ cells in both non-IRR and TBI/HSCT. Higher resting phosphorylation levels of both p38 and S6k1 were observed in CD4^+^ cells from TBI/HSCT CCS (p<0·01 and p=0·04 respectively). Peripheral blood mononuclear cells were isolated and treated as described in Figure 2 and data analysed as reported in Figure 2. **c**. DNA methylation was measured on sorted CD8^+^ T-cell at three specific CpG sites (*ASPA, ITGA2B* and *PDE4C*) and compared between the two groups, showing a significant hypermethylation on PDE4C in the TBI/HSCT group (p<0.01).

**Additional Figure 2.** **Intracellular signaling pathways involved in T-cell polarization in response to direct and indirect irradiation**. **a.** No significant effect of direct irradiation was observed on phosphorylated c Jun N terminal kinase (pJNK) and pAkt Ser473 in Jurkat cells at any time point (Day1, 14 or 28, p>0·05). **b.** No significant effect of direct irradiation was observed on the percentage of Jurkat cells producing interferon (IFN)-γ or interleukin (IL)-4 was observed at any time point (Day1, 14 or 28, p>0·05). **c.** Culture in conditioned media obtained from irradiated adipocytes did not affect pJNK in CD4^+^ cells (p>0·05) and resulted in lower pAkt Ser473 in CD4^+^ cells exposed to conditioned media from adipocytes irradiated with 6Gy (p<0·05). Cells were cultured and treated and data analysed as described in Figure 2.

**Additional Figure 3.** **Intracellular signaling pathways and polarized activation in CD8^+^ cells exposed to conditioned media from irradiated adipocytes**. **a.** Culture in conditioned media obtained from irradiated adipocytes did not affect p38 in CD8^+^ cells (p>0·05) but significantly reduced phosphorylated ribosomal protein S6 kinase 1, phosphorylated c Jun N terminal kinase and pAkt Ser473 in CD8^+^ cells exposed to conditioned media from adipocytes irradiated with 6Gy (p<0·05). **b.** Culture in conditioned media obtained from irradiated adipocytes did not affect the number of CD8^+^ cells producing interferon (IFN)-γ or interleukin (IL)-4 (p<0·05). Cells were cultured and treated and data analysed as described in Figure 3.

**Additional Table 1.** **Differentially Methylated Genes**


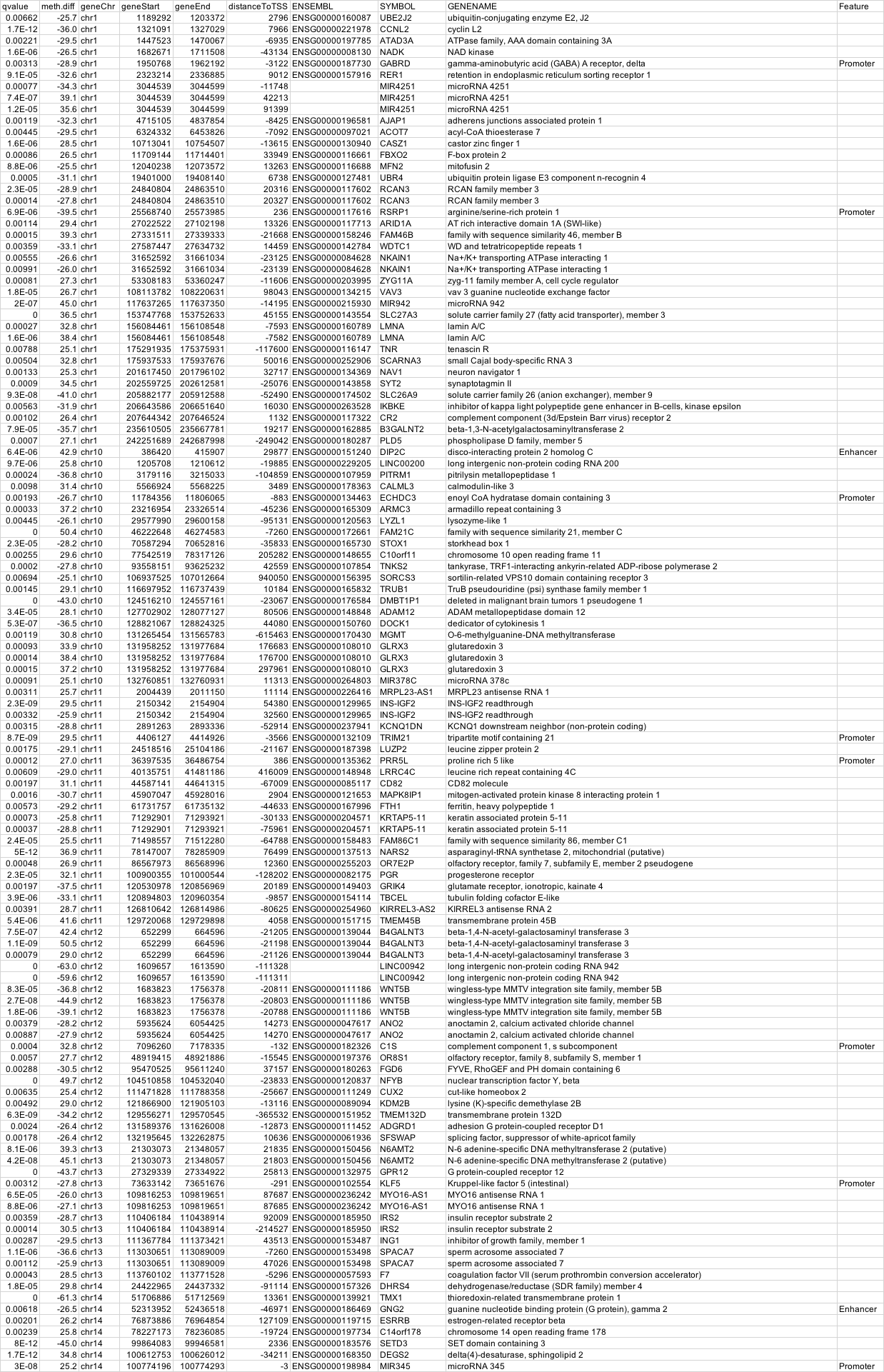


**Additional Table 1.** (continued)


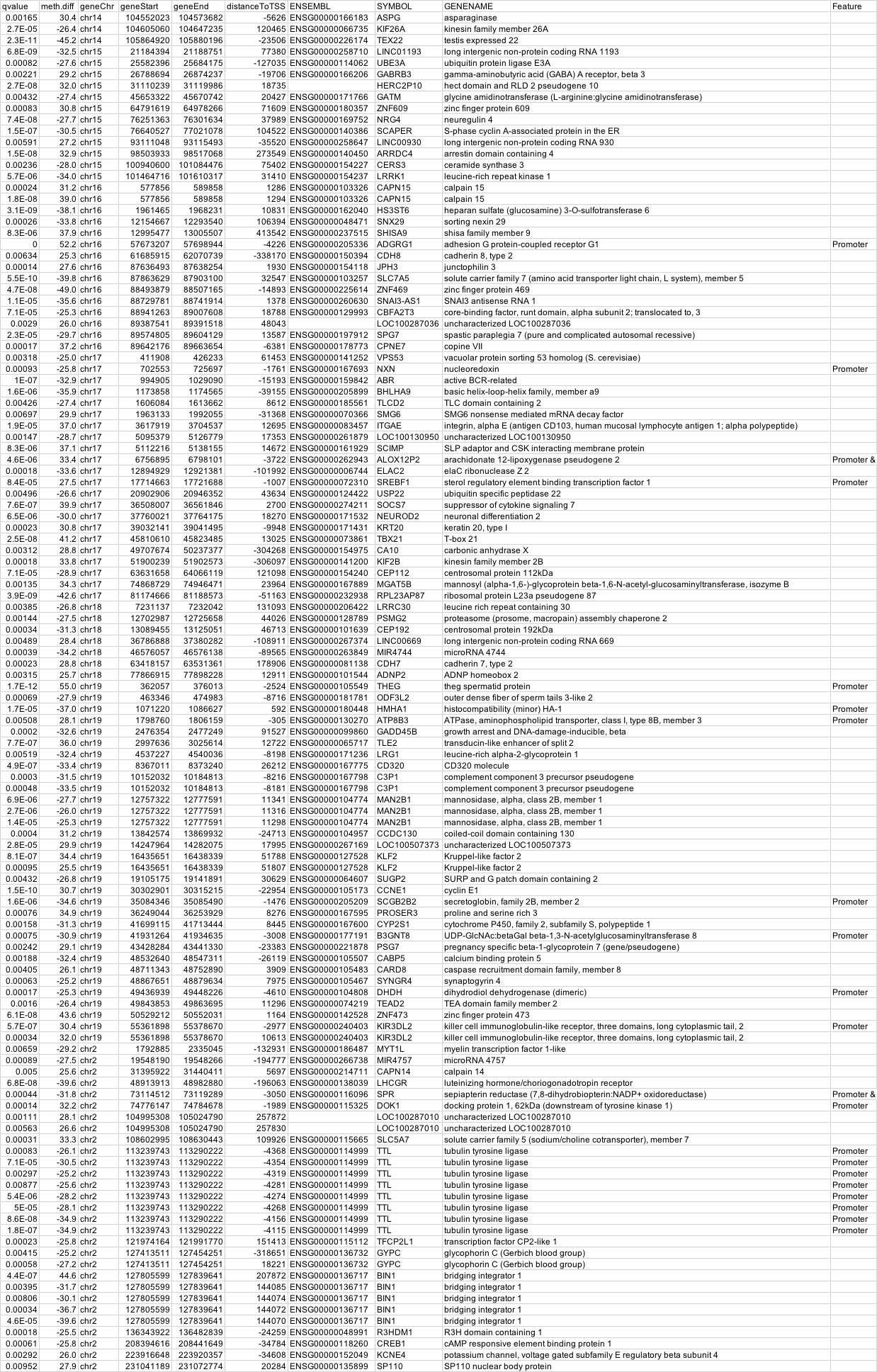


**Additional Table 1.** (continued)


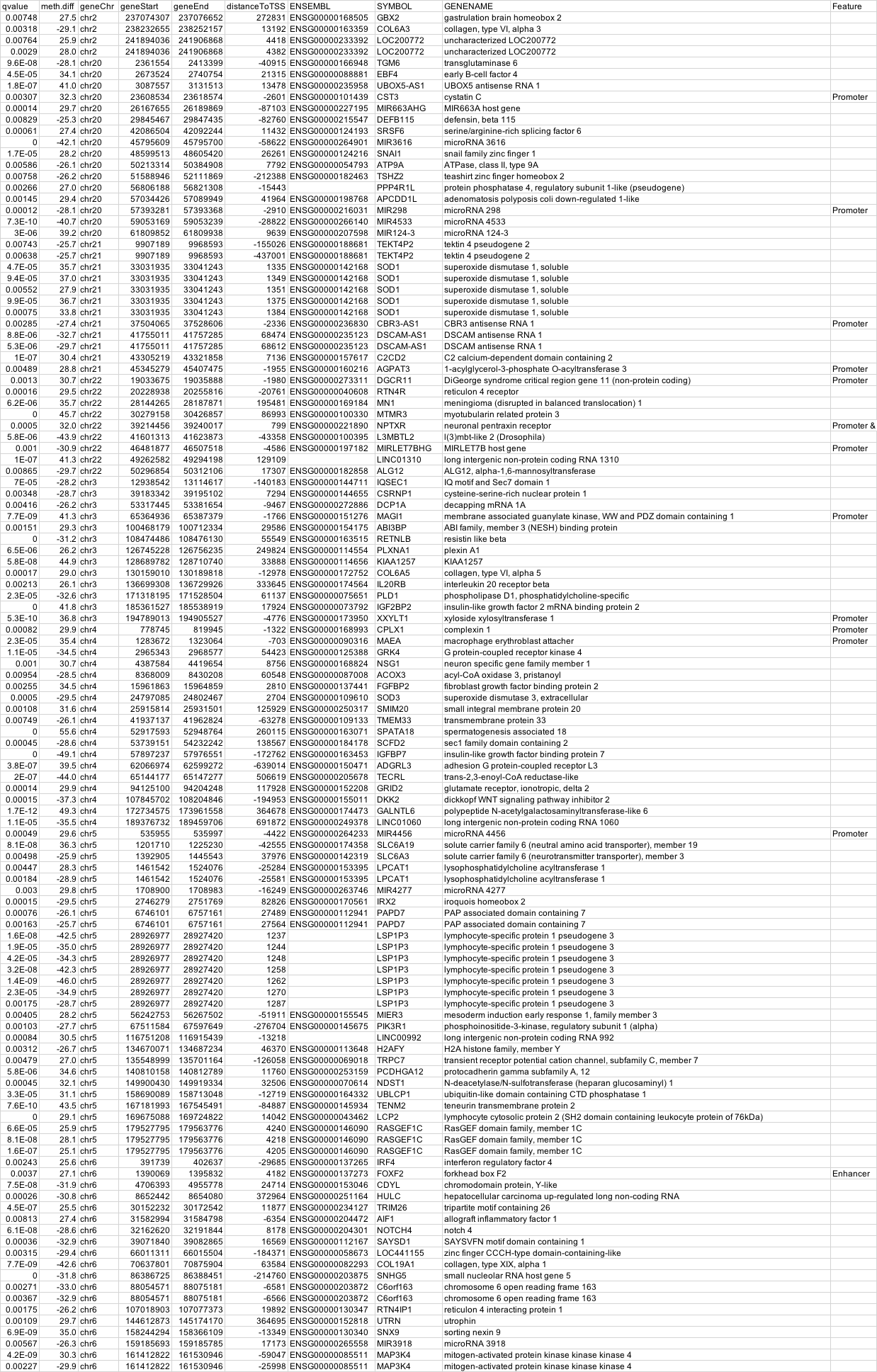


**Additional Table 1.** (continued)


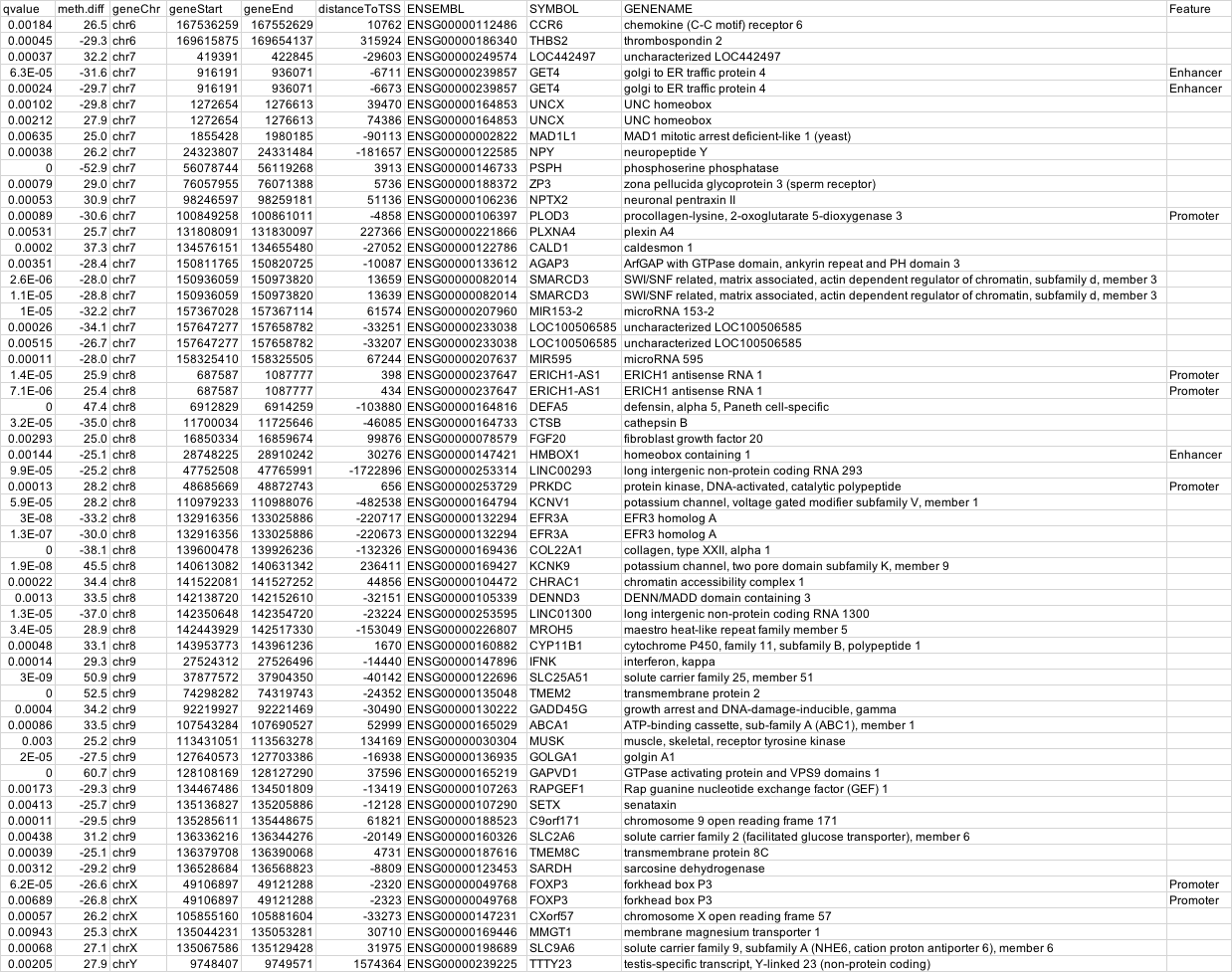


Meth diff: difference in methylation, gene chr: gene chromosome, TSS: transcription start site, ENSEMBL: Ensembl identifier

**Additional Table 2.** **Gene Ontology from differentially methylated genes**

FDR: false discovery rate.

**Additional Table 3a.** **Genes differentially expressed**

logFC: log fold change, logCPM: log count per million, FDR: false discovery rate.

**Additional Table 3b.** **Gene Ontology terms from**

**differentially expressed genes**

FDR: false discovery rate.

**Additional Table 4.**

**Additional Table 4.** (continued)

**Additional Table 4.** (continued)

**Additional Table 4.** (continued)

**Additional Table 4.** (continued)
