# Supplementary figures and images for: T cell epigenetic remodeling and accelerated epigenetic aging are linked to long-term immune alterations in childhood cancer survivors
Source: Clin Epigenetics. 2018 Nov 6;10:138. doi: 10.1186/s13148-018-0561-5 (PMC6219017; doi:10.1186/s13148-018-0561-5)

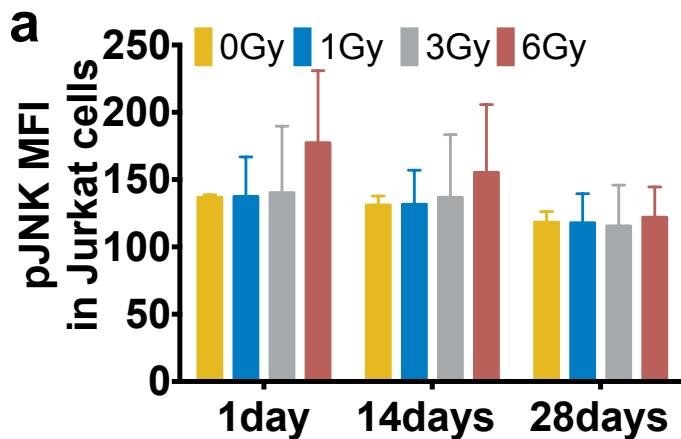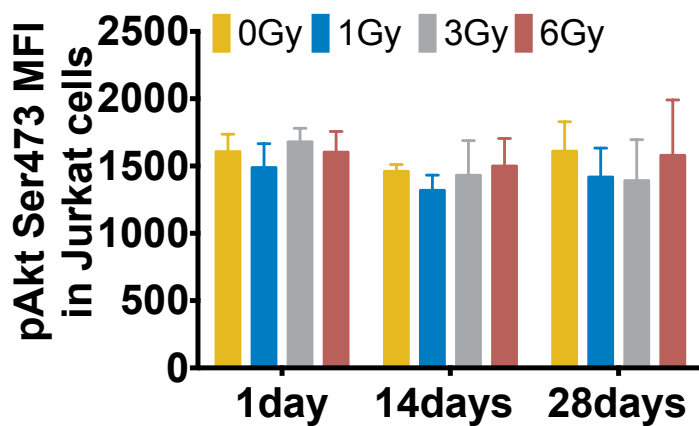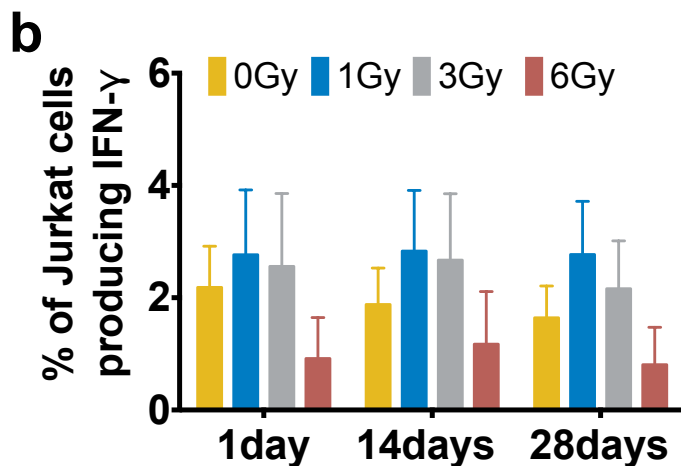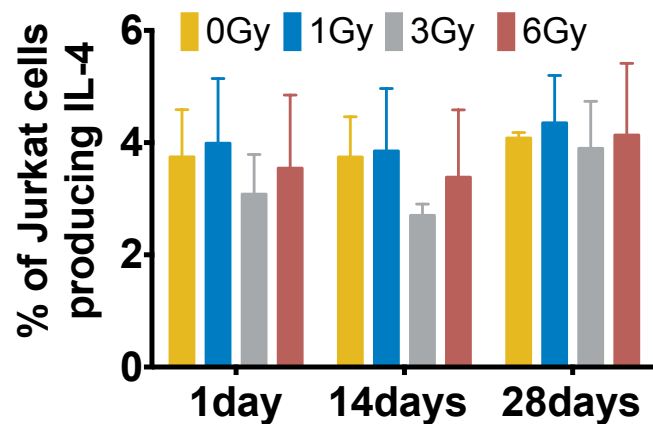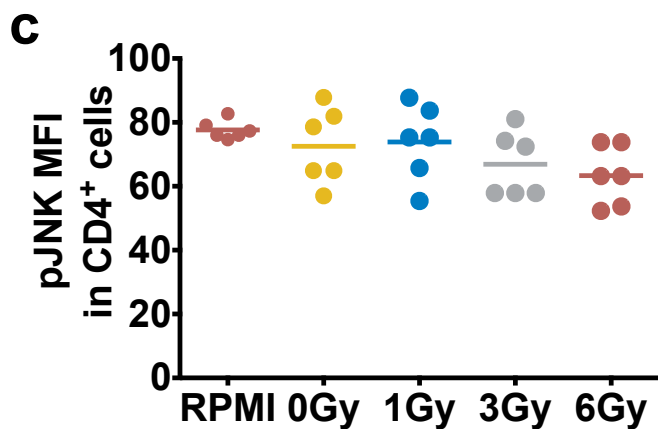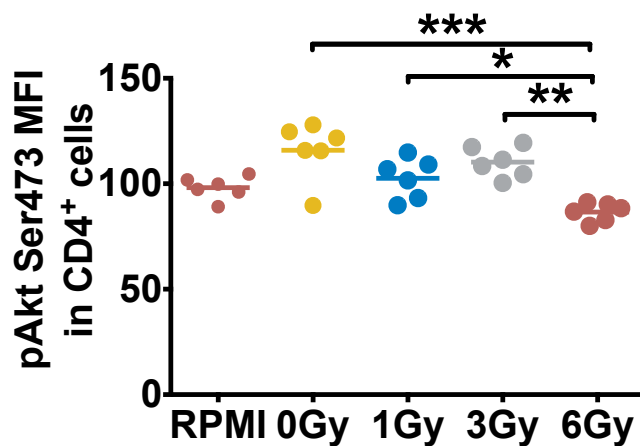

Supplement: Supplementary file 1 — Figure S1. CD8+ cells polarized activation in childhood cancer survivors (CCS). Figure S2. Intracellular signaling pathways involved in T cell polarization in response to direct and indirect irradiation. Figure S3. Intracellular signaling pathways and polarized activation in CD8+ cells exposed to conditioned media from irradiated adipocytes. Table S1. Differentially Methylated Genes. Table S2. Gene Ontology from differentially methylated genes. Table S3. a. Genes differentially expressed. b. Gene Ontology terms from differentially expressed genes. Table S4. Proteins identified in the supernatant of irradiated fibroblasts. (ZIP 4590 kb) [file 13148_2018_561_MOESM1_ESM.zip › Add_Fig2_ClinicalEpigenetics_SDaniel.pdf]

**a**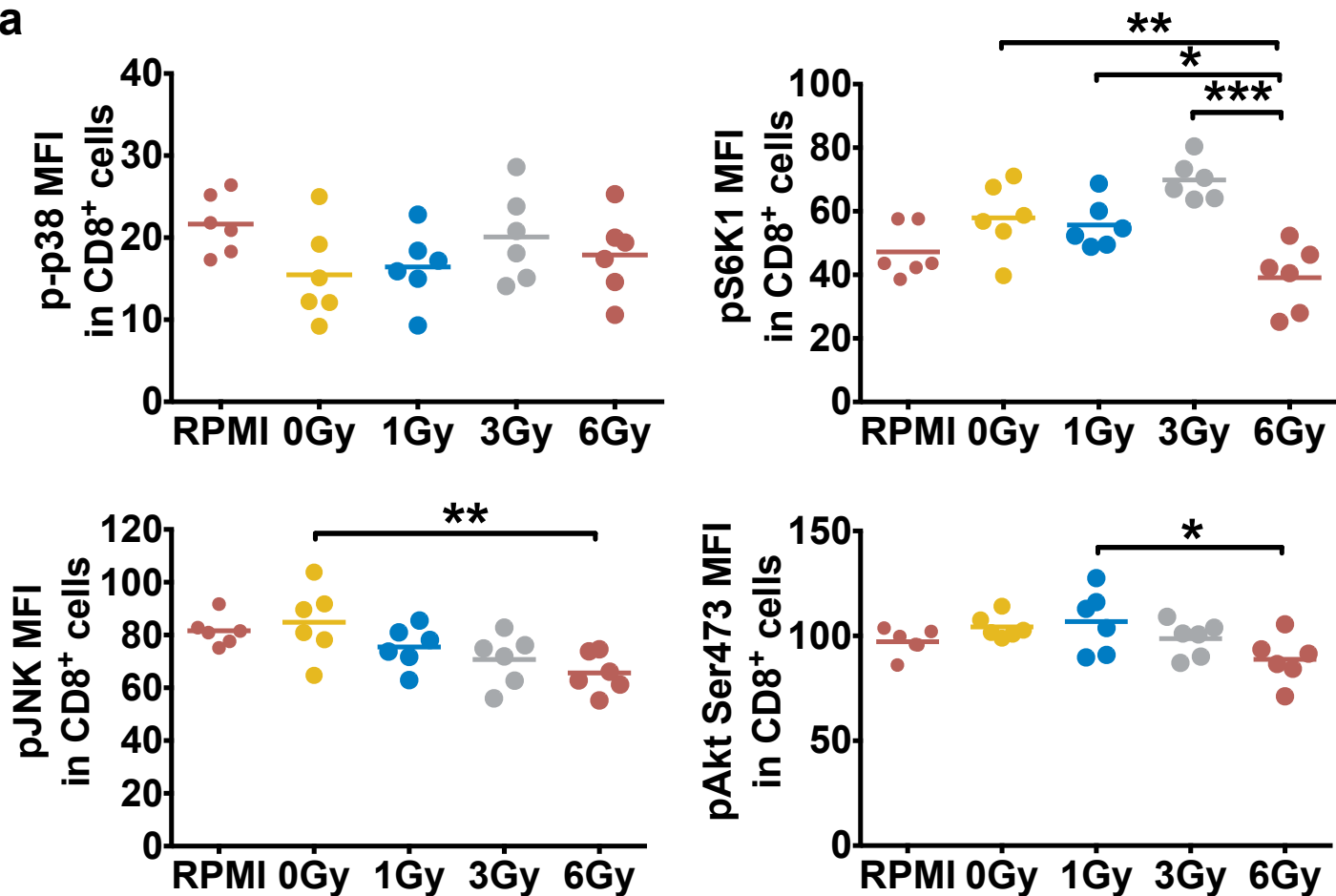**b**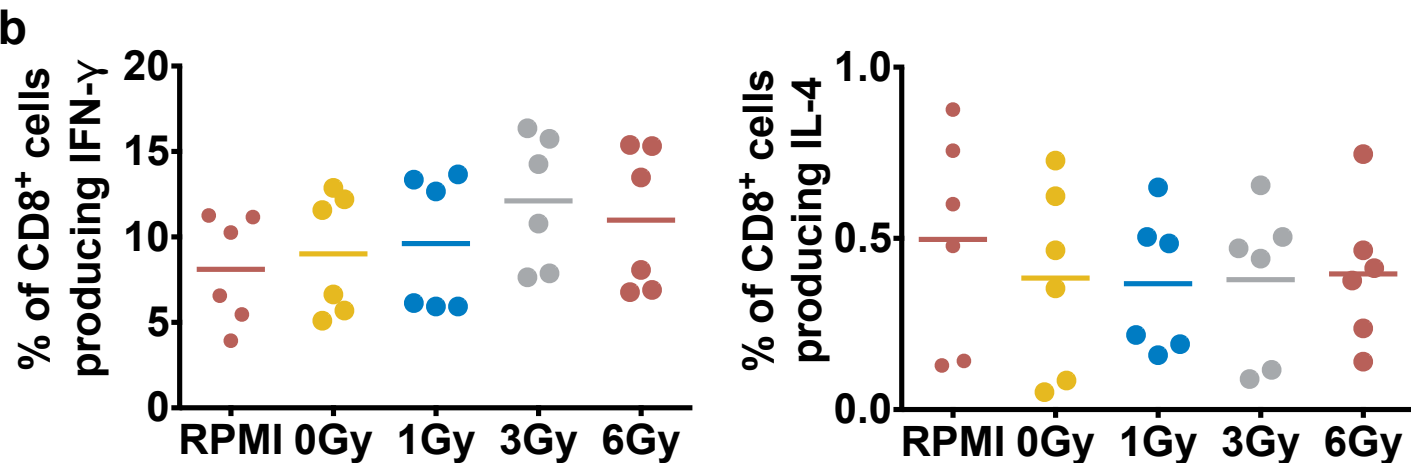

Supplement: Supplementary file 1 — Figure S1. CD8+ cells polarized activation in childhood cancer survivors (CCS). Figure S2. Intracellular signaling pathways involved in T cell polarization in response to direct and indirect irradiation. Figure S3. Intracellular signaling pathways and polarized activation in CD8+ cells exposed to conditioned media from irradiated adipocytes. Table S1. Differentially Methylated Genes. Table S2. Gene Ontology from differentially methylated genes. Table S3. a. Genes differentially expressed. b. Gene Ontology terms from differentially expressed genes. Table S4. Proteins identified in the supernatant of irradiated fibroblasts. (ZIP 4590 kb) [file 13148_2018_561_MOESM1_ESM.zip › Add_Fig3_ClinicalEpigenetics_SDaniel.pdf]

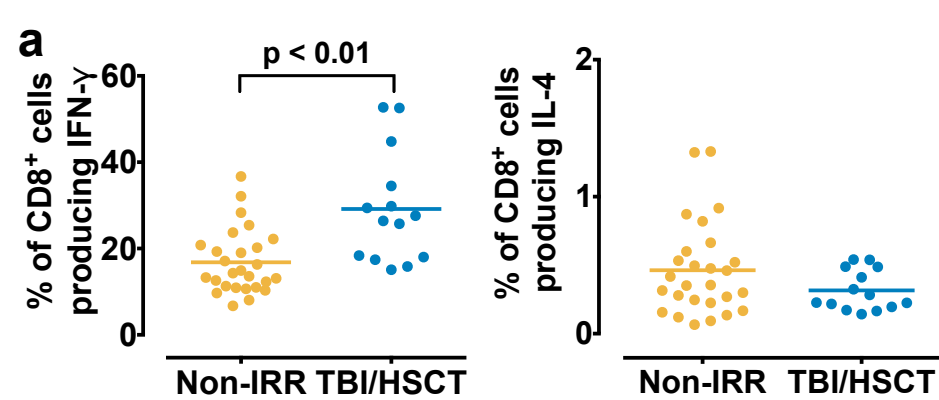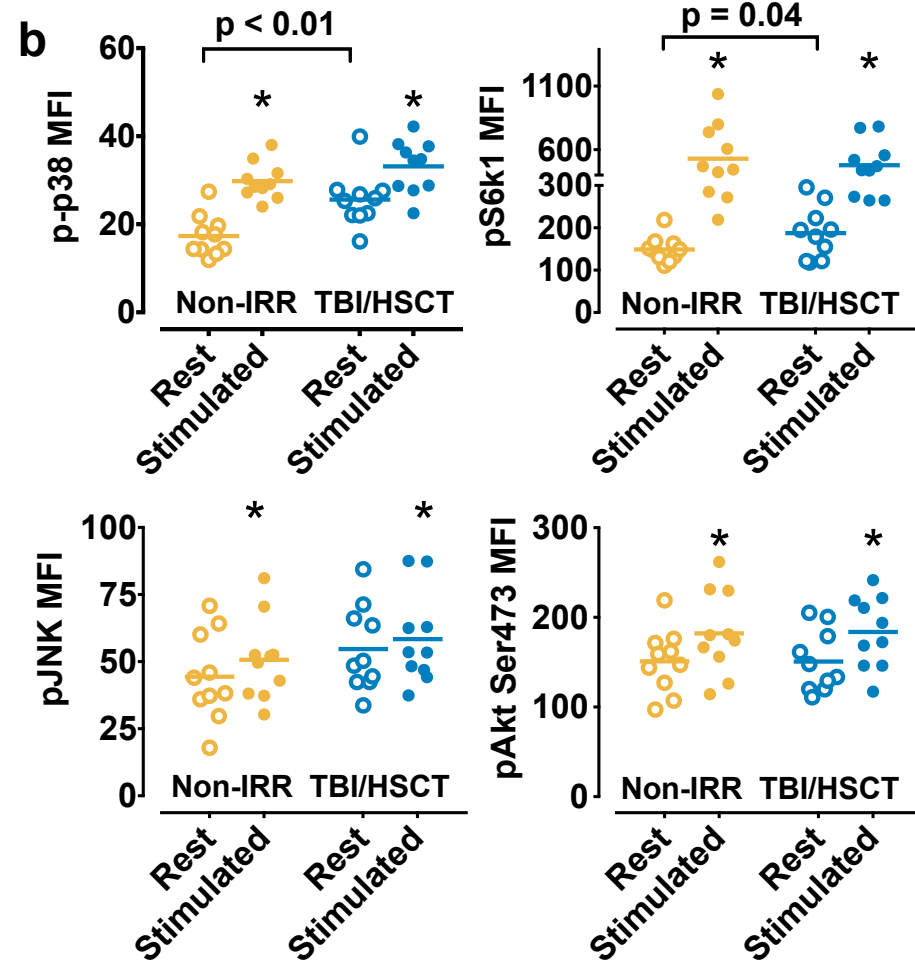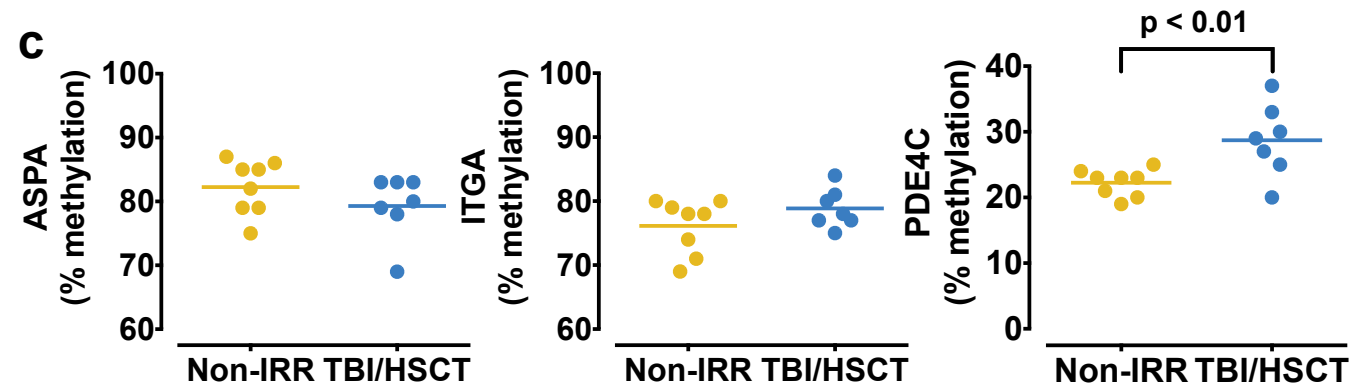

Supplement: Supplementary file 1 — Figure S1. CD8+ cells polarized activation in childhood cancer survivors (CCS). Figure S2. Intracellular signaling pathways involved in T cell polarization in response to direct and indirect irradiation. Figure S3. Intracellular signaling pathways and polarized activation in CD8+ cells exposed to conditioned media from irradiated adipocytes. Table S1. Differentially Methylated Genes. Table S2. Gene Ontology from differentially methylated genes. Table S3. a. Genes differentially expressed. b. Gene Ontology terms from differentially expressed genes. Table S4. Proteins identified in the supernatant of irradiated fibroblasts. (ZIP 4590 kb) [file 13148_2018_561_MOESM1_ESM.zip › Revised_Additional Figure 1 CLEP.pdf]
